# Supplementary material for: Speaking to a common tune: Between-speaker convergence in voice fundamental frequency in a joint speech production task
Source: PLoS One. 2020 May 4;15(5):e0232209. doi: 10.1371/journal.pone.0232209 (PMC7197779; doi:10.1371/journal.pone.0232209)
Supplement: S1 Fig — Top-left panel: global parameters with, from top to bottom: toggle Audio, set recording index, allow cross-talk, set tranformation’s phase angle (0 or π), amplitude and period. Top-right panel: visual indicators monitored during the task. Audio signal and current transformation value for participants A and B are shown on the left and right respectively, with the current turn number in the center, and the recording indicator at bottom. Bottom panel: commands to control the task. Left: pushbuttons triggering audible instructions to participants for the 4 parts of the task (silent reading, practice text, first repetition of text, second repetition text. Right: pushbuttons to initialize the task, start and stop recording in green, red and gray respectively. (PDF) [file pone.0232209.s001.pdf]

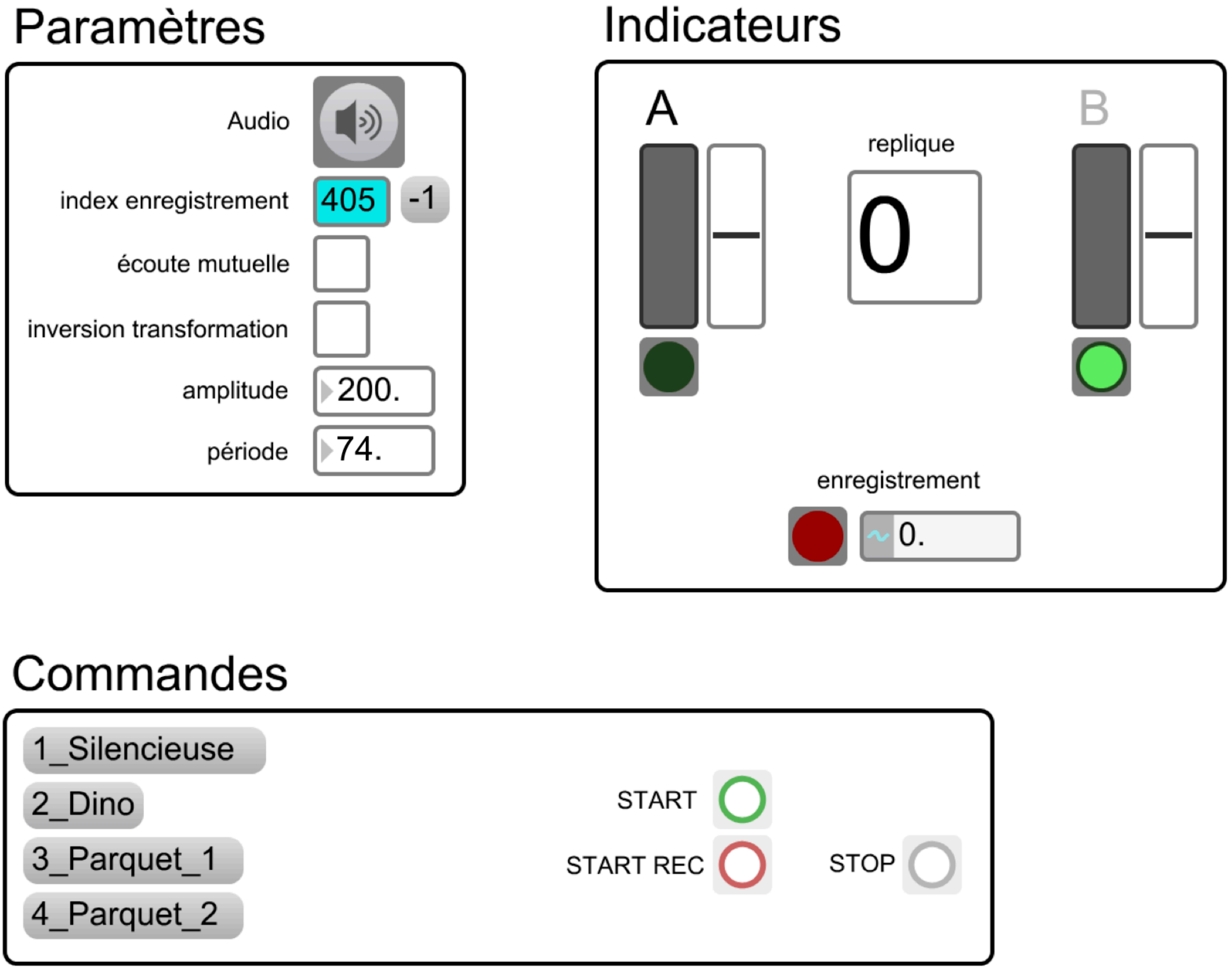
Paramètres
Audio
index enregistrement
405
-1
écoute mutuelle
inversion transformation
amplitude
200.
période
74.
Indicateurs
A
B
replique
0
enregistrement
0.
Commandes
1_Silencieuse
2_Dino
3_Parquet_1
4_Parquet_2
START
START REC
STOP
