# Supplementary material for: Speaking to a common tune: Between-speaker convergence in voice fundamental frequency in a joint speech production task
Source: PLoS One. 2020 May 4;15(5):e0232209. doi: 10.1371/journal.pone.0232209 (PMC7197779; doi:10.1371/journal.pone.0232209)
Supplement: S1 Text — For participant A (version shown here), odd-numbered turns are in boldface and are to be read aloud while even-numbered turns are in gray color and are to be listened to in the partner’s voice. For participant B (not shown), odd-numbered turns are in gray color, and even-numbered turns in boldface. Turn boundaries and turn numbers, added here for reference, were not shown in the version given to both participants. (PDF) [file pone.0232209.s002.pdf]

Supporting Information

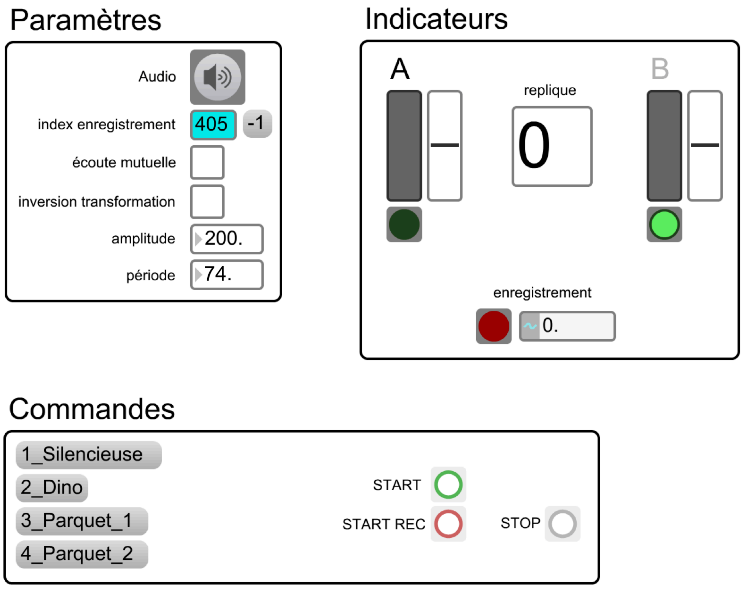

Figure 1

Interface for the voice transformation software. Top-left panel: global parameters with, from top to bottom: toggle Audio, set recording index, allow cross-talk, set tranformation's phase angle (0 or π), amplitude and period. Top-right panel: visual indicators monitored during the task. Audio signal and current transformation value for participants A and B are shown on the left and right respectively, with the current turn number in the center, and the recording indicator at bottom. Bottom panel: commands to control the task. Left: pushbuttons triggering audible instructions to participants for the 4 parts of the task (silent reading, practice text, first repetition of text, second repetition text. Right: pushbuttons to initialize the task, start and stop recording in green, red and gray respectively.

[1]**Des trois manières de poser un parquet, pose collée, flottante ou clouée,** [2]c'est la pose clouée qui est la plus traditionnelle [3]**et qui résiste le mieux au temps. Un avantage qui s'explique sans doute** [4]par l'épaisseur des lames. Voici ce qu'il faut savoir [5]**avant d'équiper son habitation avec ce revêtement de sol** [6]aux multiples atouts. Les premiers revêtements de sols en bois, de type plancher, [7]**ont été retrouvés dans le temple de Salomon. Mais il faut attendre** [8]la fin du Moyen-Age pour voir le bois se généraliser, [9]**même si on ne parle pas encore de parquets,** [10]mais de planchers menuisés. Cette appellation fait son apparition [11]**sous le règne de Louis XIV. A l'époque du Roi Soleil,** [12]les parquets sont en panneaux de chêne ouvragés, [13]**composés de motifs divers, sertis dans un cadre, puis assemblés** [14]dans un treillis de frises préparé sur le sol. Ces premiers parquets d'assemblage [15]**furent posés pour le surintendant Fouquet au château de Vaux-Le-Vicomte,** [16]et gagnèrent rapidement les sols du château de Versailles. Tous les nobles de l'époque [17]**voulurent ensuite installer dans leurs demeures un sol identique** [18]à celui de leur souverain, assurant ainsi aux parquets [19]**une publicité royale. Après la révolution, le parquet se simplifie,** [20]la lame remplace le panneau et depuis cette époque, [21]**recouvre la majorité des sols domestiques. Aujourd'hui, le terme parquet** [22]désigne un produit dont la couche d'usure ou le parement, [23]**d'une épaisseur minimale de 2 millimètres pour un produit fini, est en bois. Facile d'entretien,** [24]avec de hautes propriétés d'isolation phonique et thermique, [25]**le parquet, qui ne retient pas la poussière et n'en produit pas,** [26]est non allergique et sain. A tous ces avantages, [27]**il faut ajouter le confort, la chaleur et la durabilité** [28]qu'offre ce matériau noble et vivant. Ce type de parquet [29]**nécessite une pose très spécifique : avant la pose,** [30]il est recommandé de stocker les lames de parquet au moins 24 heures [31]**dans leur emballage d'origine, sans les ouvrir,** [32]dans la pièce où ils doivent être posés. La température ambiante de cette pièce [33]**doit être comprise entre 15 et 20 degrés. Dans le cas de pose sur un sol chauffant,** [34]un préchauffage est nécessaire. Les lames de ces parquets, [35]**plus épaisses que celles d'un parquet flottant,** [36]ne sont pas directement clouées à la chape [37]**mais sur un quadrillage de lambourdes** [38]qui mesurent au moins 3 centimètres par 4, chevillées et vissées sur la chape, [39]**et espacées l'une de l'autre de 40 centimètres, à partir de la première** [40]qui doit se trouver à 2 centimètres du mur. Ce système de pose maintient un circuit d'air [41]**sous le plancher et permet au bois de respirer,** [42]et facilite le passage de câbles ou de conduits. Ce type de parquet [43]**convient parfaitement en cas de plancher chauffant** [44]et est indispensable dans une pièce présentant des risques [45]**de remontées d'humidité. La seconde étape de la pose** [46]consiste à clouer des lames de parquet. En principe, [47]**un plancher se cloue dans la languette sur la lambourde. Si on le cloue par-dessus,** [48]en fixation apparente, c'est moins joli, [49]**mais cela va plus vite. Selon les règles de l'art du plancher cloué,** [50]le clou est inséré à la base de la languette de la lame de parquet, [51]**et se trouve masqué par la latte suivante. Chaque latte étant maintenue indépendamment** [52]et de manière ferme mais souple, lorsque le bois se rétracte en séchant, [53]**il se produit un petit espace** [54]qui crée un aspect authentique garanti. Dans toute pose de plancher, [55]**il faut toujours prévoir un jeu périphérique de 1 à 2 centimètres,** [56]sur tout le périmètre de la pièce, en laissant un espace de dilatation [57]**entre le mur et les lames que l'on peut masquer par une plinthe. Le parquet cloué,** [58]par son système de pose, surélève le niveau du sol [59]**d'une hauteur variable entre 5 et 10 centimètres. Il convient donc mieux** [60]aux constructions neuves. Si au bout de quelques années, [61]**on décide de changer de revêtement de sol,** [62]l'enlèvement du plancher cloué libérera cette profondeur de 5 à 10 centimètres, [63]**qui pourra être utilisée pour poser du carrelage,** [64]du parquet flottant ou de la moquette. En revanche, [65]**la manœuvre inverse qui consiste à remplacer ces revêtements par du plancher cloué,** [66]pose souvent un problème de fermeture des portes. En tout état de cause, [67]**la pose d'un parquet cloué ne s'improvise pas,** [68]et il est recommandé de s'adresser à une entreprise spécialisée [69]**ou un artisan parqueteur. Le chantier pour une pièce de 30 mètres carrés** [70]durera entre 2 jours et une semaine en fonction de l'habileté du poseur, [71]**du mode de pose et de la dimension des lames, selon qu'elles seront de format standard,** [72]ou en courson. Il existe une large fourchette de prix [73]**selon les différents matériaux qui s'explique par les différentes essences** [74]et les finitions. Un tarif auquel il faudra ajouter le prix de la pose.

Text 1

Text read by the participants. For participant A (version shown here), odd-numbered turns are in boldface and are to be read aloud while even-numbered turns are in gray color and are to be listened to in the partner's voice. For participant B (not shown), odd-numbered turns are in gray color, and even-numbered turns in boldface. Turn boundaries and turn numbers, added here for reference, were not shown in the version given to both participants.
