# Supplementary material for: Speaking to a common tune: Between-speaker convergence in voice fundamental frequency in a joint speech production task
Source: PLoS One. 2020 May 4;15(5):e0232209. doi: 10.1371/journal.pone.0232209 (PMC7197779; doi:10.1371/journal.pone.0232209)
Supplement: Attachment — Submitted filename: Review_PLOSOne_Dec2019.pdf [file pone.0232209.s003.pdf]

Review of ”Speaking to a common tune: between-speaker convergence in voice fundamental frequency in a joint speech production task”

This paper investigates the extent to which two speakers carrying out a collaborative reading task converge in their F0. Speakers read alternating turns in a text, but heard a version of their reading partner’s turns which had the F0 very slightly manipulated. The study finds that speakers adjusted their own F0 over time in response to the small F0 manipulations in their partner’s voice, with speakers whose F0s were already more similar to one another more closely imitating these small changes. The study is interesting and well-designed, but is in some cases not clearly communicated, and could be better-contextualized. Also, I am concerned that the statistical testing is not always appropriately carried out. Thus I recommend that the authors be asked to carry out major corrections.

Major issues:

Lack of clarity: For readers who do not use radian mathematics on a regular basis, the discussion of the stimulus modifications is extremely, and unnecessarily, confusing. Figure 2a communicates the modifications made in the stimuli, and should occur much earlier in the text (and separately from Figures 2b and 2c, which display results before the experimental design has been fully described). For ease of reading, it would be helpful if each of the relevant mathematical aspects was overly linked with its functional outcome. In some cases, a more exact specification is necessary: the text states several times that the F0 values are “shifted by” τ, the outcome of Equation 1, but it is not until p. 9 that the reader discovers that this means that τ is subtracted from the original F0 values; this information should appear much earlier.

The use of the term “amplitude” (p. 2, p. 7, p. 14) is also problematic, since amplitude is already a technical term in phonetics. It could be replaced with “maximum F0 modification” or another similar term.

Contextualization: In some cases the literature cited and the claims made appear to be somewhat orthogonal to one another. The argument that the results go against a view of convergence as “gradually build[ing] up over the course of a speech production task” (p. 15), while technically true, is taking aim at an argument which already has many counterexamples in the literature. The paper is also vague when it comes to the role of communicative function as an influence on speaker behaviour. Although “non-interactive” and “interactive” settings are discussed, it is unclear what the authors mean by this, as at least one of the studies cited as “interactive” involved a prerecorded virtual agent. Furthermore, the task differences between the “non-interactive” and “interactive” studies are substantial, and need to be considered in addition to the relative “interactivity” of the studies. This is particularly relevant since the current study is characterized as “interactive”, yet it does not involve a spontaneously developing interaction; rather, it involves the joint performance of a spoken text, in which the speakers may be more concerned with producing a consistent outcome than with communicating alignment to and with one another. The functional aspects of the particular task need to be considered more carefully. Some additional work on prosodic alignment in interactive settings includes:

Chartrand, T. L., & Bargh, J. A. (1999). The chameleon effect: the perception–behavior link and social interaction. *Journal of Personality and Social Psychology*, *76*(6), 893.

Levitan, R. (2014) Acoustic-prosodic entrainment in human-human and human-computer dialogue. Columbia University. PhD thesis.

Schweitzer, K., Walsh, M., Schweitzer, A. (2017) To See or not to See: Interlocutor Visibility and Likeability Influence Convergence in Intonation. Proc. Interspeech 2017, 919-923, DOI: 10.21437/Interspeech.2017-1248.

Zellers, M., Schweitzer, A. (2017) An Investigation of Pitch Matching Across Adjacent Turns in a Corpus of Spontaneous German. Proc. Interspeech 2017, 2336-2340, DOI: 10.21437/Interspeech.2017-811.

The authors make the somewhat odd statement that "F0 convergence across turns was found to occur in spite of the large melodic variations typically associated with reading turns". No literature is cited in support of the claim that "large melodic variations" are particularly associated with reading turns, or that these variations are larger in reading than in e.g. spontaneous conversation. If this statement is simply based on the observation of the recordings used in the study, this should be clarified.

Statistical modelling/generalizabilty: On p. 11, independent double-sided t-tests are used to determine whether δ is equal to 0. This raises two problems. First, since the F0 manipulations made are gradual and follow a pattern, δ for a particular turn is not actually independent of δ for the adjacent turns. Secondly, although multiple statistical tests are carried out, no Bonferroni adjustment (or other correction for multiple tests) is applied, or at least not reported. Thus, the results as reported are prone to false positives, and cannot be fully relied upon.

On p. 13, linear mixed-effects models are used to predict the F0 of a next turn from the F0 of a previous turn. These models include random intercepts for pairs and turns, but no random slopes, forcing the assumption that all pairs and all turns would behave in the same way (i.e. converging). However, this might not be the case. The models should be recalculated with random slopes in case different pairs or different turn combinations behave differently.

<u>Minor issues:</u>

p.3, l. 12: give full term "fundamental frequency" before starting to use the abbreviation

p. 4, ll. 47-52: the description of Goldinger's findings here is confusing; please rephrase it

p. 6, l. 103: "following responses" might be better phrased as "imitating" or "matching" responses

p. 8, Fig. 2: as discussed above, Figure 2a should come earlier, and Figures 2b and 2c later, since they contain results

p. 11, ll. 237-238: were F0 values manually corrected, or were only the limits for automatic detection manually adjusted?

p. 11, l. 241: should atan be αtan here?

p. 12, ll. 260-261: why is ϕ now calculated in turns?

p. 14, l. 309: "always lower than one-sixth" perhaps use "smaller" instead

p. 15, ll. 338-340: this sentence is confusing and should be rephrased

p. 16, ll. 377-378: the mention of joint action is interesting and could even already be mentioned in the introduction for better contextualization
