# Supplementary material for: Speaking to a common tune: Between-speaker convergence in voice fundamental frequency in a joint speech production task
Source: PLoS One. 2020 May 4;15(5):e0232209. doi: 10.1371/journal.pone.0232209 (PMC7197779; doi:10.1371/journal.pone.0232209)
Supplement: Attachment — Submitted filename: Response to Reviewers.pdf [file pone.0232209.s004.pdf]

Response to Reviewers

Manuscript number: PONE-D-19-26013

Contents

Editor **1**
Response 1 1
Response 2 2
Response 3 2
Response 4 2
Response 5 2
Response 6 2
Response 7 2
Response 8 2
Response 9 2
Response 10 2
Response 11 3
Response 12 3
Response 13 3

Reviewer #1 **3**
Response 14 3
Response 15 3
Response 16 4
Response 17 4
Response 18 4
Response 19 4
Response 20 5
Response 21 5
Response 22 5
Response 23 5
Response 24 6
Response 25 6
Response 26 6
Response 27 7
Response 28 7
Response 29 7
Response 30 8
Response 31 8
Response 32 8
Response 33 8
Response 34 8

Reviewer #2 **9**
Response 35 9
Response 36 9
Response 37 10
Response 38 11
Response 39 11
Response 40 12
Response 41 12
Response 42 12
Response 43 12
Response 44 12
Response 45 12
Response 46 12
Response 47 13
Response 48 13
Response 49 13
Response 50 13

Other comments **13**
Response 51 13

Comments from Editor

> *I have now received two clear and detailed assessments of the manuscript you submitted to PLOS One entitled "Speaking to a common tune: between-speaker convergence in voice fundamental frequency in a joint speech production task". While both reviewers consider this paper to represent an interesting contribution to the field, the first reviewer in particular raised a number of major issues regarding the description and analysis of the data. I agree with this reviewer' that the findings should be contextualized more precisely, and that the conclusions reached in some of the statistical analyses may not have been warranted. Taken together, addressing these and all other suggestions may require carrying out new analyses and reworking several sections of the text. However, I am convinced that addressing the reviewers' comments will improve the quality of your manuscript. I therefore hope you will consider resubmission. I look forward to receiving your revised manuscript.*

Response 1. We thank you and the reviewers for your comments, which we have made every effort to fully address. Below you will find our responses to each of these comments.

l. 9: "speaker's mental lexicon": Perhaps you could use 'mental sound representations' or something similar, as it is not obvious whether/how phonetic convergence may affect the structure of the lexicon beyond phonetic and phonological processing.

Response 2. We now refer to: words' spoken forms in the mental lexicon that continuously evolve throughout the speaker's lifespan.

l. 14: "indexical information associated with the speaker's age, gender, and/or emotional state": Paralinguistic uses of F0 have been claimed to play communicative roles (cf. Ohala and Gussenhoven's paralinguistic uses of their biological codes).

Response 3. "Indexical information" has been replaced by "communicative cues".

l.57. Consider dropping "have" for consistency in the use of past tenses.

Response 4. Done.

l. 85: "to what extent" this projects some kind of comparison later.

Response 5. We changed this to: we asked whether, and if so to what extent [. . .]

l.102: Consider dropping "the" in "the F0".

Response 6. Done.

l.116: "that participants read aloud alternatively": it'd be useful for the reader to have an approximate idea of how long the task was.

Response 7. We now indicate: The average duration of each repetition was 4 min and 33 s across the 32 pairs of participants. In all, the experiment lasted around 30 minutes.

l. 130: "achieve a turn". Not clear what is meant by "achieve" here.

Response 8. This was changed to: produce a turn.

l. 184: "following [13] study: Please check grammar.

Response 9. This was changed to: following Sato et al.'s [13] study.

l. 264: "7.5 turns": 75 turns

Response 10. The value of 7.5 turns for the phase angle ϕ, as estimated by fitting a sinusoidal function to the observed values for δ, was correct.

l. 303: "in the immediately preceding turn.". Since m3 performed better than m1 and m2, shouldn't turn be "preceding turns"?

Response 11. In this sentence, we refer to turn $t - 2$, the participant's preceding turn, associated with model $m2$, on the one hand, and to turn $t - 1$, the immediately preceding turn, as produced by the participant's partner, and associated by model $m1$, on the other hand. We therefore propose to keep the sentence's initial wording.

l. 306: Consider dropping the word "even".

Response 12. Done.

l. 316: "a tendency that was found to establish from": Please check grammar.

Response 13. This was changed to: a tendency that was found to establish itself from [. . .]

Comments from Reviewer #1

In this study, the authors investigate pitch adaptation, the tendency for two speakers to converge towards similar pitch patterns over the course of a conversation (in this study during a scripted dialogue). They found that speakers imitated each other's changes in F0 across turns and that speakers were both able to perceive their partner's changes in F0 and imitate these variations. The paper is clear and well written. The statistical analyses and experimental design are appropriate. The approach taken to study pitch convergence is novel and will be valuable to the expert reader. Below some suggestions for improvement of the manuscript.

Abstract :

1/ "F0, in spite of the major role played by F0": instead of F0 (parameter), maybe rather use "pitch" (perception)

Response 14. This was changed to: in spite of the major role played by pitch, F_0's perceptual correlate

2/ "we asked to what extent two speakers converge towards each other with respect to variations in F0 in a scripted dialogue.": investigate instead of ask

Response 15. Done.

Title:

3/ "Speaking to a common tune: between-speaker convergence in voice fundamental frequency in a joint speech production task" the authors may just use the term pitch convergence to avoid the need of clarifying "voice" in "voice fundamental frequency"

Response 16. We propose to keep using the term fundamental frequency as our analyses have focused on the acoustic level.

Introduction

4/ "It is thought to contribute to setting up a conversational common ground between speakers and to facilitate mutual understanding ": the authors may add here the other effect/role of convergence in strengthening relationship. This is of importance as the participants in this study knew each other prior to the experiment. It may be assumed that some form of phonetic convergence is already set in place in other contexts of social interaction they are used to engage in.

Response 17. Done.

5/ "As is well known," maybe remove not necessary

Response 18. Done.

6/ "The results, however, have shown important discrepancies both across and within studies," ok but there are some consistencies of global pitch convergence (f0 median/ range) which has been well described in the literature. The authors could here be more specific about what would have led to inconsistencies across studies; what exactly was measured of f0? what temporal span was used to assess f0 changes across studies? how much time was given to the participants to potentially measure pitch convergence? How familiar were the speakers before they engaged in the experimental task?

Response 19. This is a notoriously complex issue in the phonetic convergence literature. We have added the following elements:

It is difficult to pinpoint what may be at the origin of the disparities in the occurrence and extent of convergence effects in F_0 in the abovementioned studies, given the vast array of differences that these studies show at the methodological level. These differences include the number of model speakers (from one speaker, e.g. [4,9], to 20 speakers in [12]), the number, phonological make-up and lexical status of the items used as stimuli, and the

index employed to characterize convergence from the F_0 measures, among other features. One may note, however, that three ([5,9,13]) of the studies in which convergence in F_0 was observed appear to share one characteristic that we do not find in other studies. In [5], [9], and [13], the experimenters made F_0 in the stimuli vary in a systematic way, either through resynthesis ([5,9]) or by selection of a set of F_0 values ([13]) in the material recorded by the model speaker(s). Systematic variations in F_0 in the stimuli may have facilitated the emergence of convergence effects, compared with stimuli in which the range of F_0 variations was not controlled.

> *7/ "Our main objective was to contribute to better characterizing the size of convergence effects in F0 in both the temporal and frequency domains. More specifically, we aimed to experimentally determine whether and if so to what extent convergence in F0 between human speakers extends across turns. We also sought to establish how accurately speakers may imitate changes in their partner's F0 that are both limited in magnitude and spread over large intervals.": The objectives could come earlier in the introduction to highlight the novelty of the work. Otherwise it may not be clear to the reader in what way this study advances current knowledge and measurement of pitch convergence.*

Response 20. The objectives as stated here have been moved up to the end of Paragraph 2 in the introduction.

> *8/ "This framework could explain why speakers perform with outstanding accuracy with little training at uncommon speech tasks such as shadowing [8] or synchronous speech [35]." "with little training" could be misleading. Yes these are uncommon speech tasks and speakers are not trained for these specific tasks however the fact that they are consistently engaged in social interaction in their day-to-day activities make them expose to others' speaking styles and therefore they may engage in pitch convergence on a daily basis.*

Response 21. We have removed the phrase "with little training".

> *Materials and methods*

> *9/ The sections participants and procedure could be moved before the experimental design section to set the context for the experimental design.*

Response 22. Done.

> *10/ "communicated with each other using microphones and headphones.": for the reader's interest, the author could specify here the reason why the speakers were put in separate rooms (e.g. not to rely on non-verbal cues to communicate).*

Response 23. We have added the following: The participants' positioning in different booths ensured that each participant's voice was conveyed to the other participant through this

electronic communication channel only, and that aerial sound transmission between participants was blocked.

11/ "the participants did not notice that their partner's voice had been artificially manipulated." How did the authors control for that? Post questionnaire? Also, what was the lag between speakers' turns?

Response 24. The Results section now starts as follows: Pairs of participants accomplished the joint reading task in a smooth and fluent way, as indicated by the short lag (mean duration: 219 ms, SD: 253 ms) between each reading turn and the following one. As verified during a debriefing with the experimenter that followed the experiment, none of the participants noticed that the voice of their partner had been artificially modified.

12/ For that reason, we recruited female participants: it is important to state this in the inclusion criteria and maybe add this as a limitation to the work as you may have measured here the effect of gender (male vs. female) or gender-mixed pair (female-female vs female-male) on adaptation capacity.

Response 25. In the Participants section, we now indicate:

Whether there are variations in the amount of between-speaker phonetic convergence as a function of speaker gender has been a matter of debate. In an often-cited work, [42] found that female shadowers converged towards the model speaker to a greater extent than male shadowers. However, more recent studies with a focus on gender-related differences in phonetic convergence (e.g., [1,3,4,38,43–50]) have provided results that were inconsistent in that respect. In Pardo's seminal study [3] on phonetic convergence in conversational interactions, for example, convergence was found to be greater for male than for female speakers. In another, large-scale study, Pardo et al. [50] found no difference in the amount of phonetic convergence depending on speaker gender, whether in their conversational interaction task or in their speech shadowing task. Because gender was not a focus of interest in our study, we chose to only have female participants, as in [6,51–55], for technical reasons explained below.

13/ "We chose our sample size following [13] study, where F0 convergence effects were observed with cohorts of 24 participants exposed to F0 values varying from 196 to 296 Hz (for female 185 voices), that is, a variation of 714 cents. Given our planned variation of 400 cents, that 186 is, about half of that in [13], we estimated that we would need at least twice as many 187 participants to observe an F0 convergence effect. .. we provisioned for an additional one third of participants, resulting in planned participants." A power analysis maybe?

Response 26. We added a paragraph in the Results sections (and a reference to that paragraph in the text referenced above) to include a post-hoc evaluation of the robustness of the effect. We find that the model coefficients obtained on simulated data generated using the same noise characteristics as our initial dataset given our subject sample are significant for a very high proportion of the time (see l. 352-353), validating a-posteriori the subject

sample size.

We also moved a sentence indicating that data from 2 participants had to be discarded from l. 186 to l. 158 as we felt it was more appropriate there.

We noted that the data for these two participants had mistakenly not been removed for a subset of our previously reported analyses. As the data for this pair were incomplete (F0 was not properly detected for one recording session), removing it did not affect the results, as expected. We have updated Fig. 3 and 4 and have corrected the associated numerical values (l. 364 and 373).

> *14/ "Participants came in pairs to the lab with the requisite that pair members knew each other well and had an age difference of no more than ten years. Both the familiarity and the age difference criteria were expected to facilitate the participants' joint accomplishment of the reading task." A) This goes back to my previous comments that "little training" may not be appropriate in the context of this study, specifically when the authors ensured that the participants knew each other well to facilitate joint accomplishment;*

Response 27. The phrase "little training" has been removed.

> *B) how did you control for similar familiarity across pairs?*

Response 28. In the Participants section, we have added the following:

Fulfillment of these criteria was established by means of a questionnaire filled in by the participants prior to the experiment. Both the familiarity and age difference criteria were expected to facilitate coordination between participants in the reading task. Duration of acquaintance ranged from a number of weeks (6 pairs) to several months (14 pairs) or years (9 pairs). Age difference was lower than 3 years for most (26) pairs and did not exceed 8 years.

> *C) there may be a bit of circularity here. The results on the ability to both perceive slight changes in f0 and imitate F0 changes are to be taken in the context that the speakers were selected according to their familiarity. In this context, the results of them being able to quickly hear f0 changes and imitate are highly predictable and therefore it could be argued that the found convergence is just the result of pairing familiar individuals together.*

Response 29. In the Results section, we have added the following:

A potential limitation of our work relates to the fact that our pairs of speakers already knew each other, since the question may be raised whether familiarity between speakers may have contributed to facilitating convergence in F_0. However, in the only study known to us on the potential links between familiarity and convergence, Pardo and colleagues [57] found that perceived convergence between college roommates did not differ over the course of the academic year. Thus, the available experimental evidence does not point to

an increase in phonetic convergence with increased familiarity.

> *15/ "median F0 value in the channel of the participant that had spoken during that turn" why not looking at the range too?*

Response 30. Although we agree that it could be interesting to extend convergence measures to other parameters as well, such as F0 range, we did not have a specific hypothesis regarding potential convergence effects in F0 range with our experimental design (i.e., a narrower range or a larger range). However, we do now present a statistics on F0 variations within turns, see our Response 38.

> *Results*

> *16/ "We examined to what extent participants converged towards each other in mean F0, by calculating the correlation in mean F0 across pairs of participants. Over the entire duration of the task, this correlation was found to be significantly positive (r = 0:45; p < 0:02, see Fig 3a)". Providing that the authors measure f0median over the turn and that F0 median per turn tend to decline over the course of a paragraph, how can the authors ascertain that the speakers were adapting to each other and that the found correlation is not a by-product of global declination naturally used by both readers in the context of text reading (in this global context - larger temporal span)?*

Response 31. In this part of the analyses, mean F_0 was computed for each participant over the entire duration of the task. This should have prevented potential F_0 declination between the beginning and end of the task from having an impact on the between-participant correlation.

> *17/ "showing that co-participants who were closer to each other in mean F0 tended to more closely imitate each other's perceived shifts in F0 from one turn to the next" yes and this should be contextualised as per my previous comment of using same-gender pair and concluding on ability to adapt.*

Response 32. The potential links between gender and convergence are now evoked in the Participants section, see Response 25.

> *18/ "as soon as each speaker starts being exposed to the other speaker's voice." This could be misleading provided that the speakers are used to each other's speaking style and know each other well.*

Response 33. We have changed this to: as soon as speakers start interacting with each other.

> *19/ The authors could emphasize the novelty of their approach of pitch convergence (experimental design and measure), and may make it a study goal in order to highlight the contribution of their work to the literature.*

Response 34. Our main objectives are now exposed earlier in the paper, in the introduction's paragraph 2. In the introduction's penultimate paragraph, we also underline the innovative character and advantages of our experimental set-up.

Comments from Reviewer #2

> *This paper investigates the extent to which two speakers carrying out a collaborative reading task converge in their F0. Speakers read alternating turns in a text, but heard a version of their reading partner's turns which had the F0 very slightly manipulated. The study finds that speakers adjusted their own F0 over time in response to the small F0 manipulations in their partner's voice, with speakers whose F0s were already more similar to one another more closely imitating these small changes. The study is interesting and well-designed, but is in some cases not clearly communicated, and could be better-contextualized. Also, I am concerned that the statistical testing is not always appropriately carried out. Thus I recommend that the authors be asked to carry out major corrections.*
>
> *Major issues:*
>
> *Lack of clarity: For readers who do not use radian mathematics on a regular basis, the discussion of the stimulus modifications is extremely, and unnecessarily, confusing. Figure 2a communicates the modifications made in the stimuli, and should occur much earlier in the text (and separately from Figures 2b and 2c, which display results before the experimental design has been fully described). For ease of reading, it would be helpful if each of the relevant mathematical aspects was overly linked with its functional outcome. In some cases, a more exact specification is necessary: the text states several times that the F0 values are "shifted by" τ, the outcome of Equation 1, but it is not until p. 9 that the reader discovers that this means that τ is subtracted from the original F0 values; this information should appear much earlier.*

Response 35. The presentation of the experimental design has been clarified as follows. Initial Figure 2 has been split into a new Figure 2 and Figure 3, and placed in the Methods and Results sections respectively. The Participants and Procedure sections were moved before the Experimental section (see also our Response 22). The subtraction of τ to the original F0 values is in fact an evaluation of the quality of the transformation (we measured the difference between expected and actual values). To avoid misinterpretation of that operation as being part of the experimental manipulation, we moved that paragraph in the Results section. We therefore propose to maintain the term 'shift' to refer to the application of τ to the participants' voice as it describes unambiguously that the F0 is raised or lowered depending on the sign of the formula in Eq. 1, visually shown in Fig. 2).

> *The use of the term "amplitude" (p. 2, p. 7, p. 14) is also problematic, since amplitude is already a technical term in phonetics. It could be replaced with "maximum F0 modification" or another similar term.*

Response 36. We agree that amplitude, when used as a standalone term, is often used as a proxy for loudness or intensity in phonetics. However, it is also commonly used to refer to the height of a waveform (even in phonetics) and formalised with the variable 'A' (including in our Eq. 1). We therefore propose to continue using this term in this acception. We have however reviewed all occurrences of the term and have clarified its usage to remove any potential ambiguity (e.g., l. 223, 228: "amplitude of the transformation").

> *Contextualization: In some cases the literature cited and the claims made appear to be somewhat orthogonal to one another. The argument that the results go against a view of convergence as "gradually build[ing] up over the course of a speech production task" (p. 15), while technically true, is taking aim at an argument which already has many counterexamples in the literature. The paper is also vague when it comes to the role of communicative function as an influence on speaker behaviour. Although "non-interactive" and "interactive" settings are discussed, it is unclear what the authors mean by this, as at least one of the studies cited as "interactive" involved a prerecorded virtual agent. Furthermore, the task differences between the "non-interactive" and "interactive" studies are substantial, and need to be considered in addition to the relative "interactivity" of the studies. This is particularly relevant since the current study is characterized as "interactive", yet it does not involve a spontaneously developing interaction; rather, it involves the joint performance of a spoken text, in which the speakers may be more concerned with producing a consistent outcome than with communicating alignment to and with one another. The functional aspects of the particular task need to be considered more carefully. Some additional work on prosodic alignment in interactive settings includes:*
>
> *Chartrand, T. L., & Bargh, J. A. (1999). The chameleon effect: the perception–behavior link and social interaction. Journal of Personality and Social Psychology, 76(6), 893.*
>
> *Levitan, R. (2014) Acoustic-prosodic entrainment in human-human and human-computer dialogue. Columbia University. PhD thesis.*
>
> *Schweitzer, K., Walsh, M., Schweitzer, A. (2017) To See or not to See: Interlocutor Visibility and Likeability Influence Convergence in Intonation. Proc. Interspeech 2017, 919-923, DOI: 10.21437/Interspeech.2017-1248.*
>
> *Zellers, M., Schweitzer, A. (2017) An Investigation of Pitch Matching Across Adjacent Turns in a Corpus of Spontaneous German. Proc. Interspeech 2017, 2336-2340, DOI: 10.21437/Interspeech.2017-811.*

Response 37. We agree with the points made by the reviewer. Our goal is not to claim that ours is the first piece of work to provide a counterexample to the view of convergence as gradually building up. We wish to point out, however, that this view is well-established and used as framework in many studies on convergence.

We no longer refer to a distinction between non-interactive and interactive settings. Tasks that we presented as being conducted in an interactive setting in the first version of the manuscript, are now described as "tasks that involve pairs of participants speaking in a

turn-taking fashion".

> *The authors make the somewhat odd statement that "F0 convergence across turns was found to occur in spite of the large melodic variations typically associated with reading turns". No literature is cited in support of the claim that "large melodic variations" are particularly associated with reading turns, or that these variations are larger in reading than in e.g. spontaneous conversation. If this statement is simply based on the observation of the recordings used in the study, this should be clarified.*

Response 38. What we meant is that the melodic variations occurring naturally in the reading task are much larger than the modifications experimentally applied. We added an evaluation of the average F0 range across participants for each turn, which confirms the extent of these variations.

> *Statistical modelling/generalizabilty: On p. 11, independent double-sided t-tests are used to determine whether δ is equal to 0. This raises two problems. First, since the F0 manipulations made are gradual and follow a pattern, δ for a particular turn is not actually independent of δ for the adjacent turns. Secondly, although multiple statistical tests are carried out, no Bonferroni adjustment (or other correction for multiple tests) is applied, or at least not reported. Thus, the results as reported are prone to false positives, and cannot be fully relied upon.*

Response 39. We agree that independent t-tests were not the most convincing way to report our results, as it might indeed trigger the question of multiple comparison testing. We note however that such a correction would not apply here, as we do not have the same hypotheses for each of the comparisons, and this is what prompted our previous explanation distinguishing between beginning, middle and end portions of the turns.

In addition, our previous reporting of the results was based on a two-step sinusoidal model fitting, involving first the estimation of 2 coefficients a and b (along with significativity values), then a conversion into the final 4 coefficients of interest (C, A, ω, and ϕ). This approach did not however provide a direct estimation of the significativity of these 4 parameters.

This is why, in this revised version, we propose to abandon the independent t-tests to report the significativity of our results, and instead use the `nls()` function of the R package `stats` which provides individual estimations of coefficient significativity.

We do keep the results of the independent t-tests as a way to select turns where the convergence was highest (Fig. 4a). We added an explicit mention at l. 370 that these t-tests were uncorrected.

We also note that this modification prompted us to reverse the order of the two difference terms in the formulation of δ (Eq. 2), so that the resulting series is modeled with a sine wave with a zero-phase lag, instead of a π-phase lag when using the initial ordering of the difference terms. Fig. 3 and 4c have been modified accordingly.

In sum, we think that this new way of reporting these results is more concise, simple and convincing.

On p. 13, linear mixed-effects models are used to predict the F0 of a next turn from the F0 of a previous turn. These models include random intercepts for pairs and turns, but no random slopes, forcing the assumption that all pairs and all turns would behave in the same way (i.e. converging). However, this might not be the case. The models should be recalculated with random slopes in case different pairs or different turn combinations behave differently.

Response 40. Models have been recalculated by adding random slopes (for both predictors). The text and the table have been modified accordingly.

Minor issues:

p. 3, l. 12: give full term "fundamental frequency" before starting to use the abbreviation

Response 41. Done in both the Abstract and the Introduction sections.

p. 4, ll. 47-52: the description of Goldinger's findings here is confusing; please rephrase it

Response 42. Done.

p. 6, l. 103: "following responses" might be better phrased as "imitating" or "matching" responses

Response 43. "following responses" has been changed to "same-direction responses".

p. 8, Fig. 2: as discussed above, Figure 2a should come earlier, and Figures 2b and 2c later, since they contain results

Response 44. Done.

p. 11, ll. 237-238: were F0 values manually corrected, or were only the limits for automatic detection manually adjusted?

Response 45. Only the limits were manually adjusted, this has been been clarified.

p. 11, l. 241: should atan *be* $\alpha\mathrm{tan}$ *here?*

Response 46. This part of the text is no longer included in the manuscript.

> *p. 12, ll. 260-261: why is ϕ now calculated in turns?*

Response 47. We now report the value of ϕ both in radians, as formulated in Eq. 1, and in its equivalent in reading turns.

> *p. 14, l. 309: "always lower than one-sixth" perhaps use "smaller" instead*

Response 48. Done.

> *p. 15, ll. 338-340: this sentence is confusing and should be rephrased*

Response 49. This sentence has been rephrased to: "Another significant outcome of this work is that speakers converged to a greater extent towards each other (as measured by F0 shifts across turns) when they were already close to each other (as measured by overall proximity in mean F0)."

> *p. 16, ll. 377-378: the mention of joint action is interesting and could even already be mentioned in the introduction for better contextualization*

Response 50. The introduction now contains a mention to the joint action approach.

Other comments

> *Re: question 3 above ["*3. Have the authors made all data underlying the findings in their manuscript fully available?"*], the authors state that data will be available upon acceptance, but do not give the specified information as requested as to why this is the case.*

Response 51. We have made our dataset freely available at `https://doi.org/10.5281/zenodo.3630439`.
